# The roles of mitochondrial tRNA mutations in non-dystrophic myotonias

**DOI:** 10.1080/23802359.2020.1839364

**Published:** 2020-11-20

**Authors:** Xue-Jiao Yu, Yu Ding

**Affiliations:** a Department of Clinical Laboratory, Quzhou People’s Hospital, Quzhou, China; b Central Laboratory, Hangzhou First People’s Hospital, Zhejiang University School of Medicine, Hangzhou, China

**Keywords:** mt-tRNA mutations, phylogenetic analysis, pathogenic, NDM

## Abstract

According a recent report by Heidari et al., a mutational screening for candidate pathogenic mitochondrial tRNA (mt-tRNA) mutations were performed in 45 Iranian patients with non-dystrophic myotonia (NDM) and 70 control subjects. Through PCR amplification and direct sequence analysis, nine mt-tRNA mutations were identified: tRNA^Met^ T4454C, tRNA^Trp^ A5568G, tRNA^Cys^ T5794C, tRNA^Arg^ A10438T and T10462C, tRNA^Leu(CUN)^ A12308G, tRNA^Thr^ A15907G, A15924G and G15928A. However, through the database searches and phylogenetic conservation analysis, we noticed that the tRNA^Thr^ A15924G, G15928A and tRNA^Leu(CUN)^ A12308G mutations should be classified ‘pathogenic’. Thus, the roles of mt-tRNA mutations in clinical expression of NDM needed to be further experimentally addressed.

## Introduction

Myotonia was a genetically and heterogeneous disease due to the electrical hyper-excitability of muscle fibers (Morales and Pusch 2019). Non-dystrophic myotonia (NDM), a common type of myotonia, had been estimated to be approximately 1 in 100,000 according to a recent report (Snyder et al. 2015). To date, the etiology of NDM was still poorly understood. It was well established that high-energy consuming tissues such as muscular and nervous systems were exclusively dependent on the ATP generation by mitochondria; therefore, mitochondrial dysfunction may play an important role in NDM. In fact, mitochondrial proteome consisted of at least 1500 proteins of which 13 were encoded by the mitochondrial DNA (mtDNA) genes (Taylor et al. 2003), furthermore, mtDNA encoded 22 tRNAs and two rRNAs. Although the mitochondrial tRNA (mt-tRNA) genes comprised only a small fraction of the mitochondrial genome, however, contributed disproportionately to the etiology of mitochondrial diseases (Chinnery and Hudson 2013). To date, over 200 different pathogenic mutations had been mapped to mt-tRNA genes (http://www.mitomap.org/MITOMAP) (Ruiz-Pesini et al. 2007), emphasizing the importance of mt-tRNAs for mitochondrial function.

The clinical and molecular diagnosis of mitochondrial diseases may be achieved by mtDNA sequence analysis for known pathogenic mt-tRNA mutations. Nevertheless, a poor genotype to phenotype correlation was very common, as in the case of tRNA^Met^ T4454C (Wang et al. 2014) or tRNA^Ser(UCN)^ T7501C mutation (Ding and Huang 2016).

Most recently, Heidari et al. (2020) and colleagues investigated the relationship between mt-tRNA mutations and non-dystrophic myotonia (NDM) in a cohort of 45 Iranian patients and 70 controls. Through genetic amplification of 22 mt-tRNA genes and Sanger sequence analysis, they identified nine mt-tRNA mutations: tRNA^Met^ T4454C, tRNA^Trp^ A5568G, tRNA^Cys^ T5794C, tRNA^Arg^ A10438T and T10462C, tRNA^Leu(CUN)^ A12308G, tRNA^Thr^ A15907G, A15924G and G15928A, and regarded the tRNA^Arg^ A10438T as a potential pathogenic mutation for NDM simply because this mutation was statistically significance between NDM and control groups (*p* < 0.05). However, the pathogenicity of these mt-tRNA mutations remained mysterious. In this study, we examined the genetic susceptibility of these mt-tRNA mutations and further discussed the relationship between these mutations and clinical phenotype.

## Materials and methods

### Database searches

We carried out the literatures searches for the presence of these nine mt-tRNA mutations via Pubmed Central (https://pubmed.ncbi.nlm.nih.gov) and other public resources (Mitomap database: www.mitomap.org) (Lott et al. 2013) with the following keywords: ‘mitochondrial tRNA^Met^ T4454C mutation’; ‘mitochondrial tRNA^Trp^ A5568G mutation’; ‘mitochondrial tRNA^Cys^ T5794C mutation’; ‘mitochondrial tRNA^Arg^ A10438T mutation’; ‘mitochondrial tRNA^Arg^ T10462C mutation’; ‘mitochondrial tRNA^Leu(CUN)^ A12308G mutation’; ‘mitochondrial tRNA^Thr^ A15907G mutation’; ‘mitochondrial tRNA^Thr^ A15924G mutation; or ‘mitochondrial tRNA^Thr^ G15928A mutation’ to identify the case-control studies published to date on the association between these nine mutations and various clinical diseases.

### Phylogenetic analysis

To determine the evolutionary conservation of the candidate pathogenic mutations, a phylogenetic approach was performed. Briefly, a total of 15 organisms’ mtDNA sequences were used for this analysis. Moreover, the conservation index (CI) was compared the human mtDNA variations with other 14 species. Notably, the CI > 75% was believed to have functional potential (Levin et al. 2013).

### Structure analysis

The tRNA^Met^ T4454C, tRNA^Trp^ A5568G, tRNA^Cys^ T5794C, tRNA^Arg^ A10438T and T10462C, tRNA^Leu(CUN)^ A12308G, tRNA^Thr^ A15907G, A15924G and G15928A mutations were individually analyzed using the published secondary structures for the mt-tRNAs with the stem and loop structure (Suzuki et al. 2011).

### Determining the pathogenicity

We further utilized the updated pathogenicity scoring system to assess these mt-tRNA mutations based on the criteria that generated by Yarham et al. (2011). Notably, if the total scores of the mutation <6, it was classified as ‘neutral polymorphism’, if the scores of the mutation were 7–10, it belonged to ‘possibly pathogenic’, 11–13 points (not including evidence from single fiber, steady-state level, or trans-mitochondrial cybrid studies), it belonged to ‘probably pathogenic’; ≥11 points (including evidence from single fiber, steady-state level or trans-mitochondrial cybrid studies) it was classified as ‘definitely pathogenic’.

## Results

### Molecular features of nine mt-tRNA mutations

**Figure 1.**
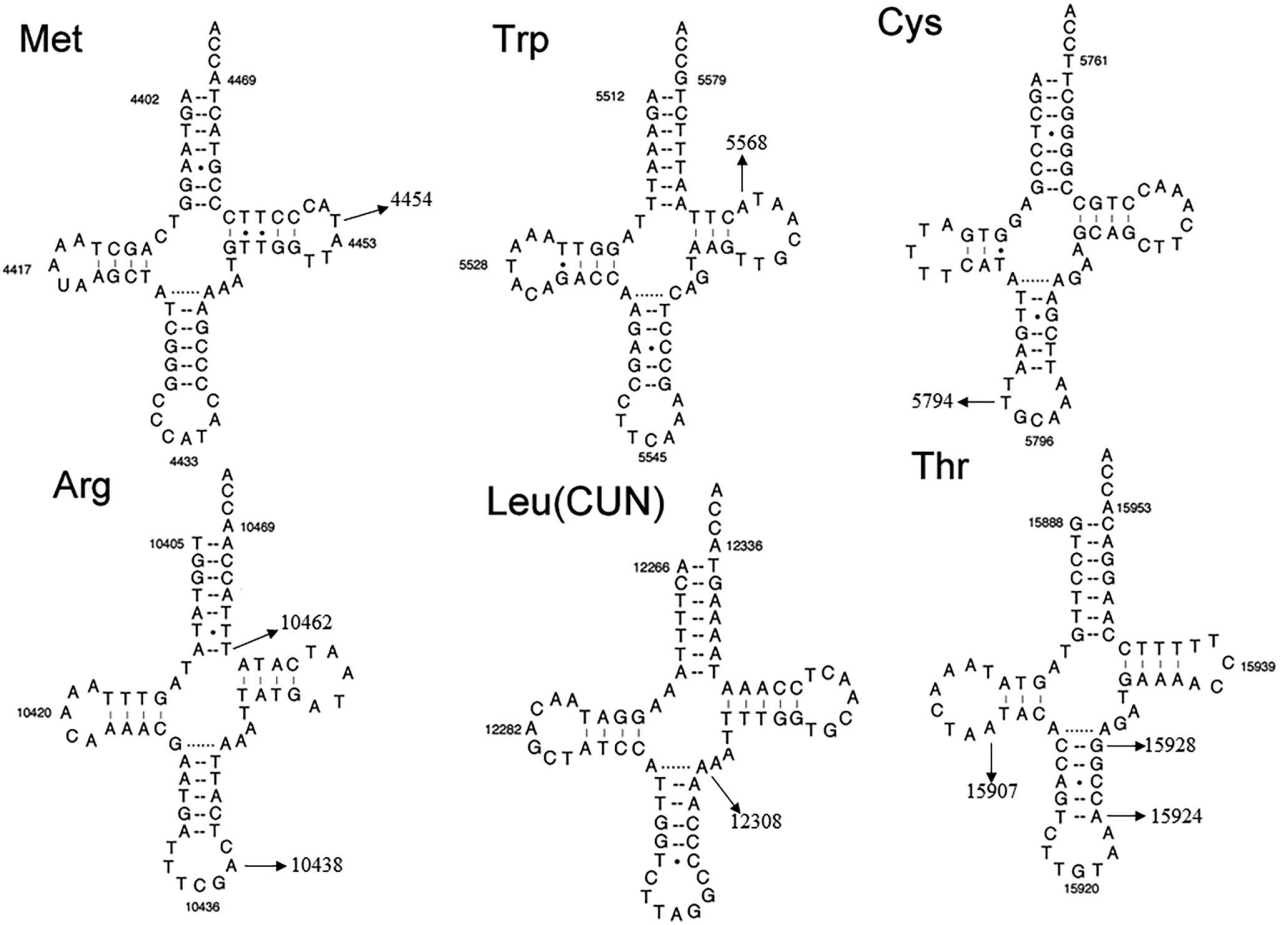
The secondary structure of nine mt-tRNA mutations, arrow indicated the mutation positions.

Heidari et al. (2020) identified nine sequence variants in mt-tRNA genes by using PCR and direct sequence. As shown in Figure 1, these mutations include the T4454C in the T-loop of tRNA^Met^ (position 58), A5568G in the T-stem of tRNA^Trp^ (position 62), T5794C in the anticodon stem of tRNA^Cys^ (position 33), T10462C (position 66) in the acceptor arm and A10438G (position 37) in the anticodon stem of tRNA^Arg^, A12308G in the variable region of tRNA^Leu(CUN)^ (position 44), A15907G (position 22) in the D-stem; A15924G (position 39) and G15928A (position 43) in the anticodon stem of tRNA^Thr^ (Florentz et al. 2003). Of these, the T10462C mutation disrupted the 7 A-66T base-pairing, the A12308G mutation created a new base-pairing (25 A-37T), while the A15924G and G15928A mutations disrupted the highly conserved base-pairings (31 T-39A and 27 C-43G), respectively.

### Analysis of CIs

**Figure 2.**
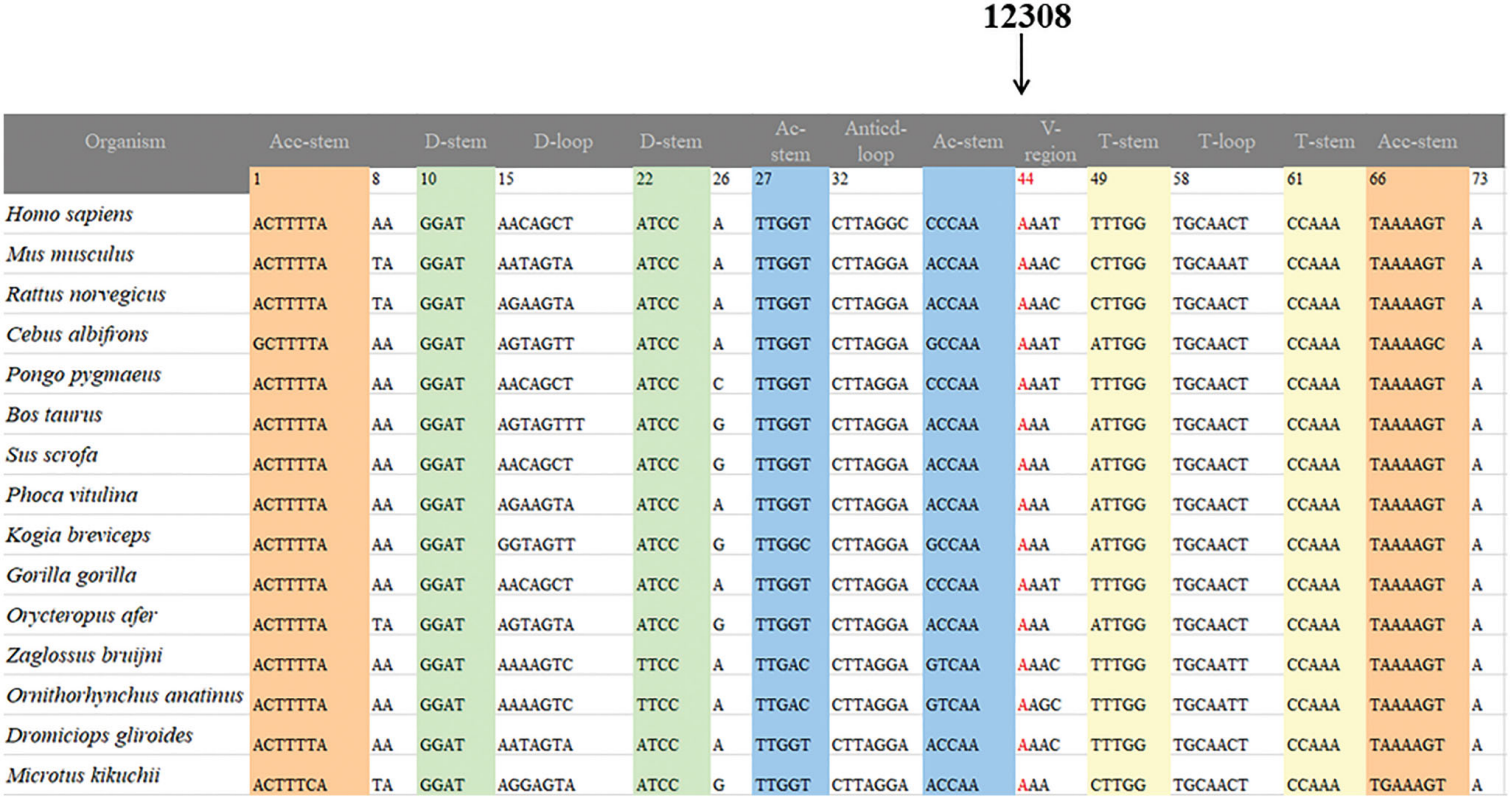
Sequence alignment of mt-tRNA^Leu(CUN)^ gene from 15 different species, arrow indicated the position 44, corresponding to the A12308G mutation.

We performed the phylogenetic conservation analysis for these nine mt-tRNA mutations, as seen Table 1 and Figure 2, we found that except for the tRNA^Arg^ A10438T, tRNA^Leu(CUN)^ A12308G, tRNA^Thr^ A15924G and G15928A mutations, other mutations are not conserved, with the CI varied from 21.1% to 82.6%, the lower level of CIs ruled out their roles in clinical expression of mitochondrial diseases.

**Table 1.** Molecular characterization of nine mt-tRNA mutations.

Gene	Mutation	Number of nucleotides in tRNA	Location in tRNA	Homoplasmy or Heteroplasmy	CI (%)	Watson-Crick base-pairing^a^	Disease association	References
tRNA^Met^	T4454C	58	T-loop	Homoplasmy	59.6		hypertension	Zhu et al. 2009
tRNA^Trp^	A5568G	62	T-stem	Homoplasmy	82.6		hearing loss	Jacobs et al. 2005
tRNA^Cys^	T5794C	33	anticodon stem	Homoplasmy	75.0		none	/
tRNA^Arg^	A10438T	37	anticodon stem	Heteroplasmy	100		progressive encephalopathy	Uusimaa et al. 2004
tRNA^Arg^	T10462C	66	acceptor arm	Homoplasmy	21.1	A-T↓	none	/
tRNA^Leu(CUN)^	A12308G	44	variable region	Heteroplasmy	100	A-T↑	chronic progressive external ophthalmoplegia; stroke; cardiomyopathy; breast cancer risk	Zifa et al. 2008; Pulkes et al. 2000; Finnila et al. 2001; Covarrubias et al. 2008; van den Ouweland et al. 1992
tRNA^Thr^	A15907G	22	D-stem	Homoplasmy	65.4		none	/
tRNA^Thr^	A15924G	39	anticodon stem	Heteroplasmy	100	T-A↓	lethal infantile mitochondrial myopathy; fatal infantile respiratory enzyme deficiency	Brown et al. 1992; Yoon et al. 1991
tRNA^Thr^	G15928A	43	anticodon stem	Heteroplasmy	100	C-G↓	multiple sclerosis; Parkinson's disease	Mayr-Wohlfart et al. 1996, 1997; Simon et al. 2000

^a^ Classic Watson–Crick (WC) base pair: created (↑) or disrupted (↓).

### Determining the pathogenicity

According to the updated pathogenicity scoring system (Yarham et al. 2011), we noticed that the total scores of mt-tRNA^Leu(CUN)^ A12308G, mt-tRNA^Thr^ A15924G and G15928A mutations were 15, 13 and 10 points, respectively, belonging to ‘definitely pathogenic’ and ‘possibly pathogenic’. Similarly, the total scores of T4454C, A5568G, T5794C, A10438T, T10462C and A15907G mutations were 4,4,2,4,4 and 2 points, respectively, suggesting that they belong to ‘neutral polymorphisms’ at this stage (Table 2).

**Table 2.** The pathogenicity scoring system for A12308G, A15924G and G15928A mutations.

Scoring criteria	A12308G	Score/20	A15924G	Score/20	G15928A	Score/20	Classification
More than one independent report	Yes	2	Yes	2	Yes	2	
Evolutionary conservation of the base pair	No changes	2	No changes	2	No changes	2	≤6 points: neutral polymorphisms;
Variant heteroplasmy	Yes	2	Yes	2	Yes	2	
Segregation of the mutation with disease	Yes	2	Yes	2	Yes	2	7–10 points: possibly pathogenic;
Histochemical evidence of mitochondrial disease	No	0	No	0	No	0	
Biochemical defect in complex I, III or IV	No	0	No	0	No	0	
Evidence of mutation segregation with biochemical defect from single-fiber studies	Yes	5	No	0	No	0	11–13 points (not including evidence from single fiber, steady-state level or trans-mitochondrial cybrid studies): probably pathogenic
Mutant mt-tRNA steady-state level or evidence of pathogenicity in trans-mitochondrial cybrid studies	Weak evidence	2	Strong evidence	5	Weak evidence	2	≥11 points (including evidence from single fiber, steady-state level or trans-mitochondrial cybrid studies): definitely pathogenic
Maximum score	Definitely pathogenic	15	Definitely pathogenic	13	Possibly pathogenic	10	

## Discussion

Mitochondrion played a critical role in cellular energy production (Chatterjee et al. 2011), in particular, mt-tRNA mutations were being increasingly recognized as important causes for disease, such mutations can result in transcriptional and translational defects and consequently mitochondrial respiratory chain dysfunction (Servidei 2003). However, it should be noted that some mutations in mt-tRNA genes cause devastating disease, whereas others had no clinical consequences (McFarland et al. 2004).

In the present study, we reassessed the roles of nine mt-tRNA mutations in the phenotypic manifestation of NDM. Among these sequence alternations, the T4454C mutation was located at position 58 in the T-loop of tRNA^Met^, nucleotide at that position was not well conserved and may not have functional impact on mitochondrial translation (Wang et al. 2014). Moreover, the tRNA^Trp^ A5568G mutation had been reported to be associated with hearing loss (Jacobs et al. 2005), however, no functional analysis was performed in cybrid cells containing this mutation, therefore, the role of A5568G mutation remained controversial. While the homoplasmic T5794C variant occurred at position 33 in the anticodon stem of tRNA^Cys^, the A10438T mutation was localized at highly conserved position in the anticodon stem of tRNA^Arg^. Notably, four mutations affected the Watson-Crick base-pairings: the tRNA^Arg^ T10462C disrupted the 7 A-66T base-pairing, the tRNA^Leu(CUN)^ A12308G created a conserved 25 A-37T base-pairing, whereas the A15924G mutation abolished the 31 T-39A base-pairing, while the G15928A mutation disrupted the 27 C-43G base-pairing (Table 1 and Figure 1). In addition, by literature searching, we noticed that the tRNA^Leu(CUN)^ A12308G mutation had been reported to be associated with CPEO, stroke and breast cancer risk (Pulkes et al. 2000; van den Ouweland et al. 1992; Covarrubias et al. 2008). While the tRNA^Thr^ A15924G mutation had been regarded as a risk factor for lethal infantile mitochondrial myopathy (LIMM) and fatal infantile respiratory enzyme deficiency (Brown et al. 1992; Yoon et al. 1991). Furthermore, the heteroplasmic tRNA^Thr^ G15928A mutation was believed to play an important role in multiple sclerosis (MS) and Parkinson’s Disease (PD) (Mayr-Wohlfart et al. 1996; 1997; Simon et al. 2000). Thus, we supposed that the A12308G, A15924G and G15928A mutations may lead to a failure in mt-tRNAs metabolism, and consequently result the impairment in mitochondrial protein synthesis (Fox 2012). In fact, the pathogenicity scoring system indicated that besides the A12308G, A15924G and G15928A mutations, others should be regarded as ‘neutral polymorphisms’ (Yarham et al. 2011).

According to these observations, we believed that the possible molecular mechanism underlying the A12308G, A15924G and G15928A mutations in clinical expression of NDM may be as follows, first of all, these mutations altered the secondary structure and affected the steady-state levels of corresponding tRNAs, subsequently, these mutations may lead to the failure in tRNA metabolism such as CCA addition, aminoacylation, or defects in tRNA modifications. As a result, mitochondrial protein synthesis may be affected by these events and the respiratory chain functions impaired, thus, the ATP declined and ROS increased, which led to mitochondrial dysfunction that was involved in the pathogenesis of NDM.

Most recently, several studies had been reported on the associations between mt-tRNA mutations and mitochondrial disorders, as in the cases of T4454C variant and hypertension (Wang et al. 2014), the C15891T variant and Leber’s Hereditary Optic Neuropathy (LHON) (Jiang et al. 2016), the tRNA^Phe^ C628T variant and hearing loss (Zhu et al. 2015). Although we believed that mt-tRNA mutations played important roles in NDM, a call for more carefully reassessment of the dataset seemed necessary. The main limitation of the current study was the lack of functional analysis for these pathogenic mutations; further studies were needed to verify this conclusion.

## Data Availability

Data sharing is not applicable to this article as no new data were created or analyzed in this study.
